# Vericiguat for heart failure with reduced ejection fraction on quadruple therapy without recent worsening heart failure: an exploratory real-world cohort study

**DOI:** 10.1186/s12872-026-06319-1

**Published:** 2026-07-21

**Authors:** Peng Han, Yuan Ning, Bohui Zhang, Xi Zhang, Che Wang, Tiantong Yu, Yan Chen, Boda Zhu, Youhu Chen, Genrui Chen, Shuai Zhao, Chengxiang Li, Yan Chen, Kun Lian

**Affiliations:** 1 Department of Cardiology, Xijing Hospital, Air Force Medical University, 169 West Changle Road, Xi’an, Shaanxi 710032 People’s Republic of China; 2 The 981th Hospital of the Joint Logistics Support Force, Chengde, Hebei People’s Republic of China; 3 School of Medicine, Northwest University, Xi’an, Shaanxi People’s Republic Of China; 4 School of Clinical Medicine, Xi’an Medical University, Xi’an, Shaanxi People’s Republic of China; 5 School of Public Health, Shaanxi University of Chinese Medicine, Xi’an, Shaanxi People’s Republic of China; 6 Department of Cardiology, Xi’an Honghui Hospital, Xi’an, Shaanxi People’s Republic of China; 7 Emergency Department, The Xijing 986 Hospital of Air Force Medical University, Xi’an, Shaanxi People’s Republic of China; 8 Harbin, People’s Republic of China; 9 Department of Cardiology, The First Affiliated Hospital of Xi’an Jiao Tong University, Xi’an, Shaanxi People’s Republic of China; 10 Air Force Hospital of Western Theater Command, Chengdu, Sichuan People’s Republic of China; 11 No.971 Hospital of the PLA Navy, 7 Shandong Road, Qingdao, Shandong 266071 People’s Republic of China

**Keywords:** Vericiguat, Chronic heart failure with reduced ejection fraction, Guideline-directed medical therapy, Propensity score matching, Quality of life

## Abstract

**Background:**

Heart failure with reduced ejection fraction (HFrEF) patients remain at substantial risk despite quadruple guideline-directed medical therapy (GDMT [ARNI + SGLT2 inhibitor + β-blocker + MRA]). Data on vericiguat in stable HFrEF patients without recent worsening heart failure receiving contemporary quadruple GDMT are limited. This observational study aimed to explore the efficacy and safety of vericiguat in this population.

**Methods:**

A total of 450 adult patients with LVEF < 45% were enrolled (200 received quadruple GDMT plus vericiguat, 250 received quadruple GDMT alone). After 1:1 propensity score matching (PSM), 144 well-matched pairs were identified. The primary outcome was the composite of cardiovascular death or first HF hospitalization within 12 months. Secondary outcomes included changes in NYHA functional class, KCCQ-12 scores, and occurrence of hypotension. Due to the non-randomized design, all efficacy findings except the primary composite endpoint are considered exploratory. Trial Registration: ChiCTR, ChiCTR2500095278. Registered 4 January 2025. Retrospectively registered.

**Results:**

Over 12 months, the primary composite endpoint occurred in 5.56% of the vericiguat group versus 9.03% of the control group (*P* = 0.365; HR 0.616, 95% CI 0.255–1.486). No statistically significant differences were observed for cardiovascular death or all-cause mortality. Exploratory analyses showed that the vericiguat group had higher KCCQ-12 Overall Summary Scores (91.61 ± 9.56 vs. 87.93 ± 11.25, *P* = 0.003) and a greater proportion achieving a ≥ 5-point improvement (94.44% vs. 81.25%, *P* = 0.001). NYHA functional class distribution also differed between groups (*P* = 0.029). Hypotension rates were similar between groups.

**Conclusion:**

Among HFrEF patients without recent worsening, vericiguat did not significantly reduce the primary composite endpoint at 12 months. Exploratory observations suggest potential associations with improved quality of life and functional class without increased hypotension risk. These hypothesis‑generating findings require confirmation in adequately powered randomized controlled trials.

**Supplementary Information:**

The online version contains supplementary material available at https://doi.org/10.1186/s12872-026-06319-1.

## Background

Heart failure with reduced ejection fraction (HFrEF) has a poor long-term prognosis, with high rates of rehospitalization and mortality, 5-year all-cause mortality exceeding 75% after hospitalization [1, 2]. Each exacerbation drives irreversible disease progression, underscoring the need for early, aggressive pharmacotherapy to mitigate morbidity and mortality. Thus, early and aggressive pharmacological intervention is essential to slow disease progression and improve outcomes.

Guideline-directed medical therapy (GDMT) comprising angiotensin receptor–neprilysin inhibitor (ARNI) or angiotensin-converting enzyme inhibitor (ACEI)/angiotensin II receptor blocker (ARB), sodium-glucose cotransporter-2 inhibitor (SGLT2i), beta-blocker (BB) and mineralocorticoid receptor antagonist (MRA) forms the foundation of HFrEF management and significantly improves clinical outcomes [3]. Yet even with this quadruple therapy, many patients still show adverse events. As an example, 14.3% of patients experienced worsening HF or cardiovascular death within 18.2 months in the DAPA-HF trial [4].

Soluble guanylate cyclase (sGC) stimulators enhance cyclic guanosine monophosphate (cGMP) production, improving cardiac and vascular function [5]. The sGC stimulator vericiguat has been shown to reduce the composite risk of cardiovascular death and HF hospitalization in high-risk HFrEF patients with a recent acute worsening event [6, 7], leading current international guidelines to recommend its addition to standard GDMT in this patient population [8–10].

However, evidence for vericiguat efficacy primarily derived from populations at high risk due to recent decompensation. Data obtained in HFrEF patients without recent worsening HF, and particularly on health-related quality of life, are limited. Although the VICTOR trial was the first phase Ⅲ study to focus on chronic, non-acute HFrEF, background use of ARNI and SGLT2i was low [11, 12], leaving a gap in evidence for vericiguat administration in combination with contemporary standard quadruple GDMT in HFrEF without recent worsening HF.

This study therefore aimed to explore the clinical outcomes and patient‑reported measures associated with vericiguat in combination with standard quadruple GDMT in this understudied patient population.

## Methods

### Study design and population

This real-world study was a prospective cohort registry based on a single center. We consecutively enrolled 450 adult patients diagnosed with HF with left ventricular ejection fraction (LVEF) below 45%. Based on clinical assessment by attending physicians, 250 patients were administered standard quadruple GDMT consisting of ARNI, SGLT2i, BB and MRA, and were assigned to the control group. The remaining 200 received the same quadruple regimen with the addition of vericiguat and were assigned to the vericiguat group.

Inclusion criteria were: (1) age ≥ 18 years; (2) New York Heart Association (NYHA) functional class [13] Ⅱ-Ⅳ, with LVEF below 45% confirmed by echocardiography within 6 months; (3) standard quadruple GDMT alone or combined with vericiguat; (4) Signed informed consent and willingness to complete scheduled follow-up. Exclusion criteria were: (1) hemodynamic instability or intravenous HF therapy within 24 h before enrollment; (2) HF hospitalization or clinical evidence of acute decompensation within the past 30 days; (3) current use of or anticipated need for long-acting nitrates, nitric oxide (NO) donors, other sGC stimulators, or phosphodiesterase-5 (PDE-5) inhibitors during the study; (4) symptomatic bradycardia or second/third-degree atrioventricular block without a permanent pacemaker; (5) prior heart transplantation/left ventricular assist device (LVAD) implantation, or planned within 12 months; (6) eGFR < 30 mL/min/1.73 m² or Child-Pugh class B/C; (7) pregnancy or lactating women; (8) conditions interfering with clinical assessment/study compliance; (9) expected survival < 1 year; (10) known hypersensitivity or contraindication to any active ingredient or excipient of the study drugs. Treatment allocation was based on physician assessment; therefore, this is an observational, non‑randomized cohort study. 

### Ethical approval and trial registration

The study protocol was approved by the Ethics Committee of the First Affiliated Hospital of Air Force Medical University (Approval No.: KY20242169-F-1). This prospective observational cohort study was conducted after obtaining ethical approval. Due to administrative and procedural delays, this study was retrospectively registered with the China Clinical Trial Registration Center on January 4, 2025 (ChiCTR2500095278). Written informed consent was obtained from all participants prior to enrollment. The study adhered to the Declaration of Helsinki and followed the STrengthening the Reporting of OBservational studies in Epidemiology (STROBE) guidelines for cohort studies.

### Data collection and definitions

All patients underwent a 12-month follow-up period following treatment initiation. The primary endpoint was a composite of cardiovascular death or first HF hospitalization within 12 months. Secondary endpoints comprised changes in NYHA functional class and Kansas City Cardiomyopathy Questionnaire-12 (KCCQ-12) scores [14]. Safety was evaluated based on the incidence of drug-related adverse events.

Baseline data were collected at the initial visit and included demographic characteristics (age, sex), body mass index (BMI), vital signs (blood pressure and heart rate), NYHA functional class, and medical history (coronary artery disease, atrial fibrillation, hypertension, hyperlipidemia, diabetes mellitus, previous myocardial infarction [MI], previous percutaneous coronary intervention [PCI], previous coronary artery bypass grafting [CABG], and previous implantable cardiovertor-defibrillator [ICD] implantation). Laboratory parameters comprised serum creatinine (Scr), estimated glomerular filtration rate (eGFR), and N-terminal pro–B-type natriuretic peptide (NT-proBNP). Echocardiographic measurements included left ventricular end-diastolic volume (LVEDV), left ventricular end-systolic volume (LVESV), and LVEF. Additionally, all patients completed the KCCQ-12 questionnaire to assess baseline health-related quality of life.

At the 12-month follow-up, NYHA class and KCCQ-12 scores were reassessed through inpatient assessments, outpatient visits or telephone interviews. Vital status and occurrence of primary endpoint events (cardiovascular death and HF hospitalization) were documented. All drug-related adverse events were systematically recorded throughout the study period.

Baseline was defined as the date of treatment initiation. Exacerbation of HF was defined as a requirement for intravenous diuretic therapy within 3 months or HF hospitalization within 6 months [6]. The primary outcome was defined as a composite clinical endpoint occurring within 12 months, namely cardiovascular death or first HF hospitalization. Hypotension was defined as a systolic blood pressure < 90 mmHg at rest. Symptomatic hypotension was defined as a state of low blood pressure accompanied by related symptoms such as dizziness, fatigue, etc. Asymptomatic hypotension was defined as a measured blood pressure below the normal standard but without any clinical symptoms or discomfort caused by the low blood pressure.

### Statistical analysis

Categorical variables were presented as n (%). Continuous variables were expressed as mean ± standard deviation (SD) deviation if normally distributed, or as median (interquartile range) if non-normally distributed. Between-group comparisons of categorical variables were performed using the chi-square (χ²) test; the Fisher’s exact test was used when expected cell frequencies were below 5 or in case of small sample size. For continuous variables, independent samples t-tests were applied for normally distributed data, while the Mann-Whitney U test was used for non-normally distributed data. To minimize selection bias between the control and vericiguat groups, propensity score matching (PSM) was performed to minimize selection bias between the control and vericiguat groups. A 1:1 sequential nearest-neighbor matching algorithm was applied without replacement, using a caliper width of 0.02 standard deviations of the logit of the propensity score. The propensity score was estimated based on the following baseline covariates: blood pressure, heart rate, history of coronary heart disease, diabetes mellitus, previous MI, previous PCI, NYHA functional class, KCCQ-12 score, and use of oral diuretics at baseline. Covariate balance before and after matching was assessed using standardized mean differences (SMDs), with an SMD < 0.1 considered indicative of adequate balance, while 0.1 ≤ SMD < 0.2 represented acceptable covariate balance. Matching was implemented with the MatchIt package in R.

In the matched cohort, binary outcomes were analyzed using the McNemar test, and ordinal outcomes were compared using the paired Wilcoxon signed-rank test. For time-to-event outcomes, survival curves were estimated using the Kaplan-Meier method, and between-group differences were assessed using the stratified log-rank test. For the KCCQ-12 scores, paired t-tests were used to compare between-group differences at 12-month follow-up, as well as to compare the magnitude of change from baseline between matched pairs.

For the analysis of continuous endpoints, missing follow-up data due to non-cardiovascular death were handled using multiple imputation. For patients who experienced cardiovascular death, follow-up indices were assigned the worst possible values (worst-rank imputation). A two-sided p-value < 0.05 was considered statistically significant. All statistical analyses were performed using R software (version 4.3.0) and SPSS (version 25.0).

As this was an exploratory real-world cohort, the sample size was determined by consecutive enrollment. The potential insufficient statistical power associated with this sample size is comprehensively discussed in the Discussion section.

## Results

### Baseline characteristics before PSM

**Fig. 1:**
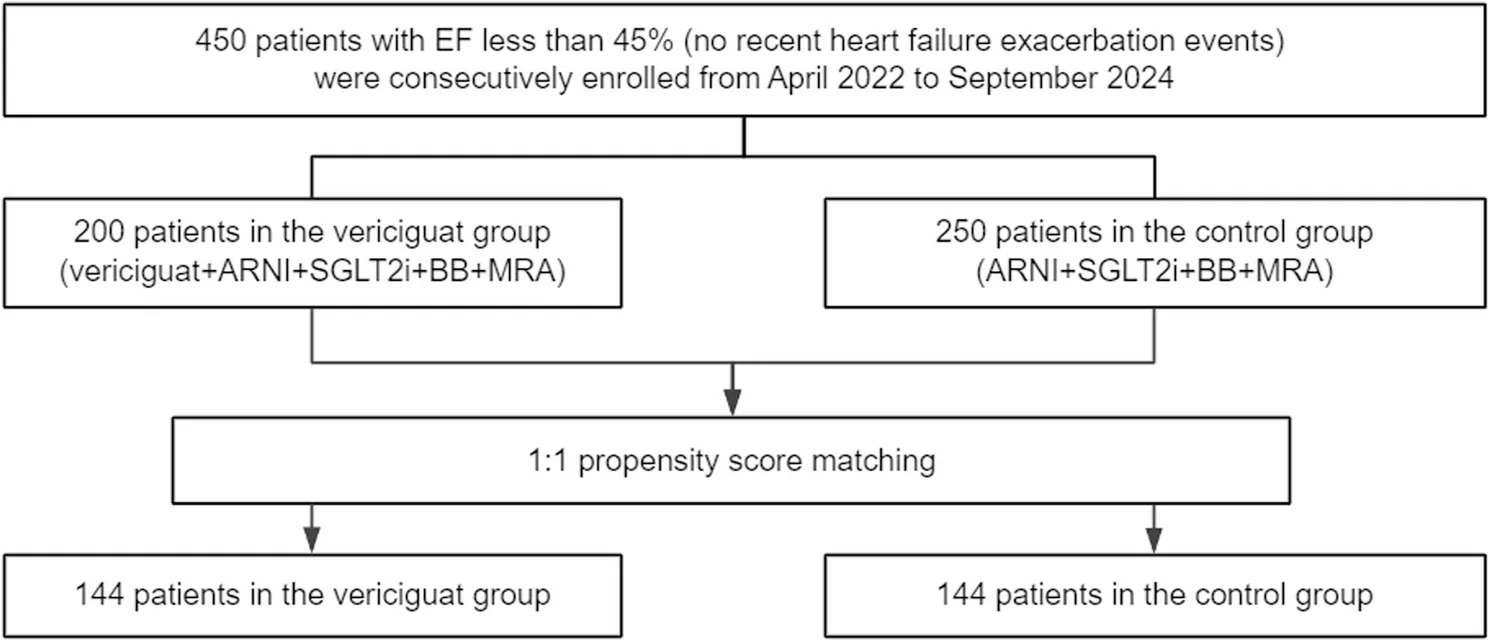
Study flowchart. (ARNI=angiotensin receptor–neprilysin inhibitor, SGLT2i=sodium-glucose cotransporter-2 inhibitor, BB=beta-blocker, MRA=mineralocorticoid receptor antagonist)

A total of 450 patients were included (200 vericiguat, 250 control). All completed 12-month follow-up; questionnaire data were missing for 7 (3.50%) and 8 (3.20%) patients, respectively. Follow-up began at the initial treatment visit. Both groups showed high adherence, with all patients reaching the target or maximum tolerated dose within 4 weeks and no discontinuations due to symptomatic hypotension or other reasons. Before PSM, significant between-group differences existed. The control group had higher rates of coronary heart disease (72.80% vs. 45.00%), hypertension (46.00% vs. 36.00%), diabetes mellitus (35.60% vs. 26.50%), prior MI (39.20% vs. 15.50%), prior PCI (26.00% vs. 14.00%), oral diuretic use (58.80% vs. 37.00%), and digoxin use (22.00% vs. 14.00%) (all *P* < 0.05). NYHA functional class distribution also differed (*P* = 0.009): the control group had more class Ⅱ (51.60% vs. 38.00%) and less class Ⅲ (55.00% vs. 41.60%). Control group had higher SBP (122.71 ± 20.81 vs. 118.43 ± 16.79 mmHg, *P* = 0.016), DBP (77.11 ± 14.28 vs. 74.17 ± 11.41 mmHg, *P* = 0.016), heart rate (77.90 ± 12.14 vs. 74.19 ± 11.11 bpm, *P* = 0.001), and KCCQ-12 Symptom Frequency Scores (83.15 vs. 77.48, *P* < 0.001). LVEF was balanced (35.30 ± 5.88 vs. 35.08 ± 6.41, *P* = 0.699). No significant differences were observed in age, sex, smoking, atrial fibrillation, BMI, NT-proBNP, renal function, or other KCCQ-12 domains (all *P* > 0.05) (Fig. 1; Table 1).

**Table 1:** Baseline clinical characteristics before PSM

	Vericiguat	Control	*P*
*n*	200	250	
Age, years	56.80 ± 12.34	59.02 ± 12.08	0.056
Male, *n*%	152 (76.00%)	197 (78.80%)	0.480
Smoking, *n*%	57 (28.50%)	86 (34.40%)	0.187
Coronary heart disease, *n*%	90 (45.00%)	182 (72.80%)	< 0.001
Atrial fibrillation, *n*%	11 (5.50%)	14 (5.60%)	1.000
Hypertension, *n*%	72 (36.00%)	115 (46.00%)	0.035
Hyperlipidemia, *n*%	47 (23.50%)	60 (24.00%)	0.912
Diabetes mellitus, *n*%	53 (26.50%)	89 (35.60%)	0.042
Previous MI, *n*%	31(15.50%)	98 (39.20%)	< 0.001
Previous PCI, *n*%	28 (14.00%)	65 (26.00%)	0.002
Previous CABG, *n*%	0	5 (2.00%)	0.069
Previous ICD implantation, *n*%	0	1 (0.40%)	1.000
Systolic blood pressure, mmHg	118.43 ± 16.79	122.71 ± 20.81	0.016
Diastolic blood pressure, mmHg	74.17 ± 11.41	77.11 ± 14.28	0.016
Heart rate, bpm	74.19 ± 11.11	77.90 ± 12.14	0.001
BMI	24.46 ± 4.05	24.55 ± 3.38	0.799
NYHA functional classification			0.009
Ⅱ, *n*%	76 (38.00%)	129 (51.60%)	
Ⅲ, *n*%	110 (55.00%)	104 (41.60%)	
Ⅳ, *n*%	14 (7.00%)	17 (6.80%)	
KCCQ-12 Overall Summary Score	65.86 ± 12.20	67.80 ± 16.88	0.157
KCCQ-12 Physical Limitation Score	69.24 ± 14.72	68.77 ± 18.49	0.765
KCCQ-12 Symptom Frequency Score	77.48 ± 15.37	83.15 ± 11.92	< 0.001
KCCQ-12 Quality of Life Score	56.12 ± 18.72	55.16 ± 22.98	0.625
KCCQ-12 Social Limitation Score	60.59 ± 24.82	64.13 ± 26.48	0.145
LVEDV, mL	188.70 ± 59.29	190.37 ± 195.13	0.898
LVESV, mL	126.67 ± 87.34	114.20 ± 47.28	0.070
LVEF, %	35.08 ± 6.41	35.30 ± 5.88	0.699
LVEF ≤ 40%, %	157 (78.50%)	200 (80.00%)	0.726
NT-proBNP, pg/mL	1432.37(418.88,3724.86)	1587.00(584.70,3704.02)	0.298
Creatinine, µmol/L	81.78 ± 14.05	82.17 ± 14.53	0.777
eGFR, mL/min/1.73 m²	102.36 ± 14.74	103.45 ± 12.08	0.401
Oral diuretics, *n*%	74 (37.00%)	147 (58.80%)	< 0.001
Digoxin, *n*%	28 (14.00%)	55 (22.00%)	0.037

### Primary clinical outcomes before PSM

**Fig. 2:**
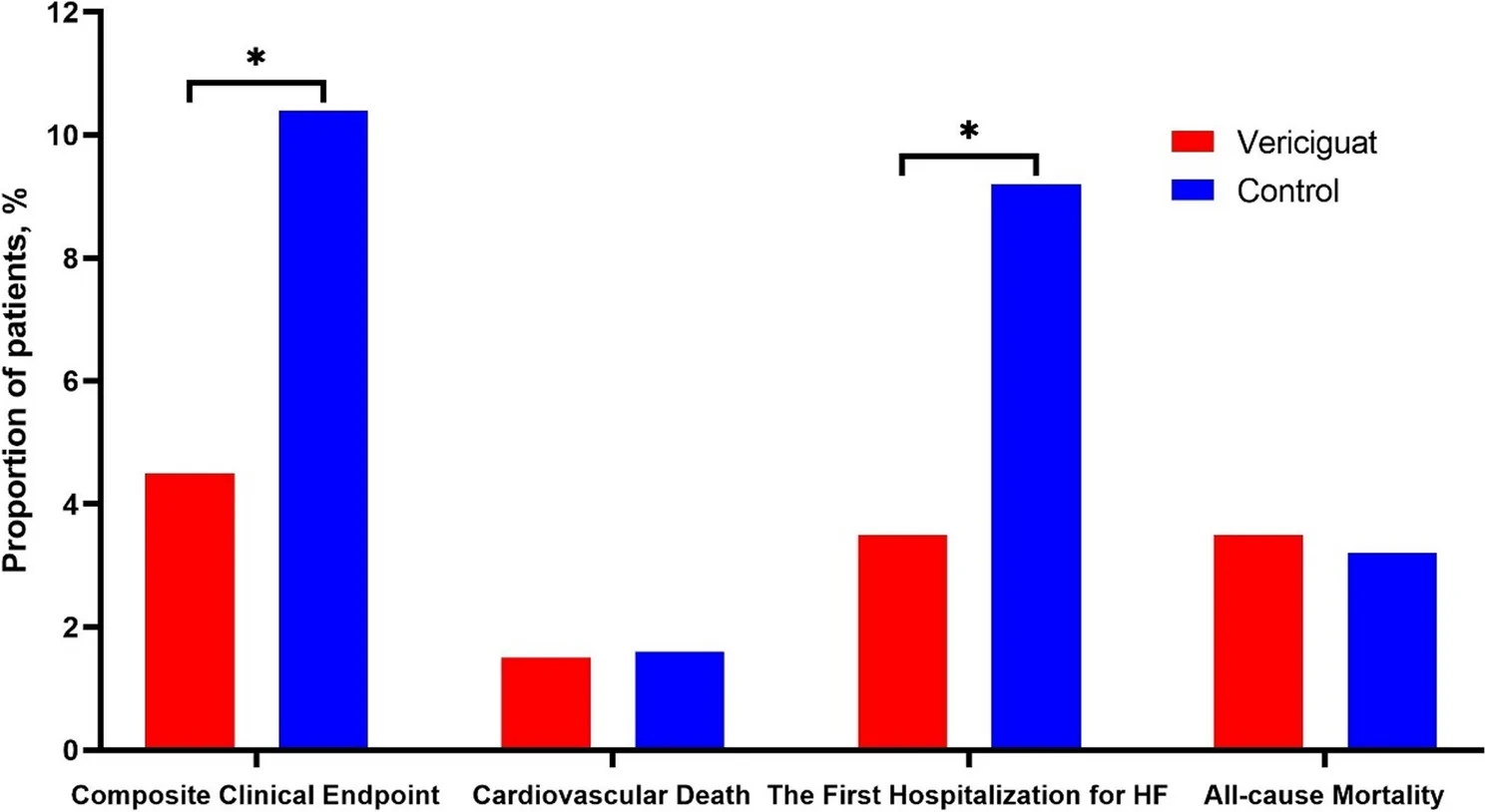
Clinical endpoints at 12-month before PSM. (*P < 0.05. Using chi square test, Fisher’s exact test was used when the expected cell frequency was below 5 or the sample size was small)

Over 12 months, the composite endpoint of cardiovascular death or first HF hospitalization occurred in 9 vericiguat patients versus 26 controls (4.50% vs. 10.40%, *P* = 0.022). First HF hospitalization rates were 3.50% versus 9.20% (*P* = 0.021). Cardiovascular death rates were 1.50% versus 1.60% (*P* = 1.000), and all-cause mortality rates were 3.50% versus 3.20% (*P* = 1.000) (Fig. 2).

### Baseline characteristics in the PSM cohort

**Fig. 3:**
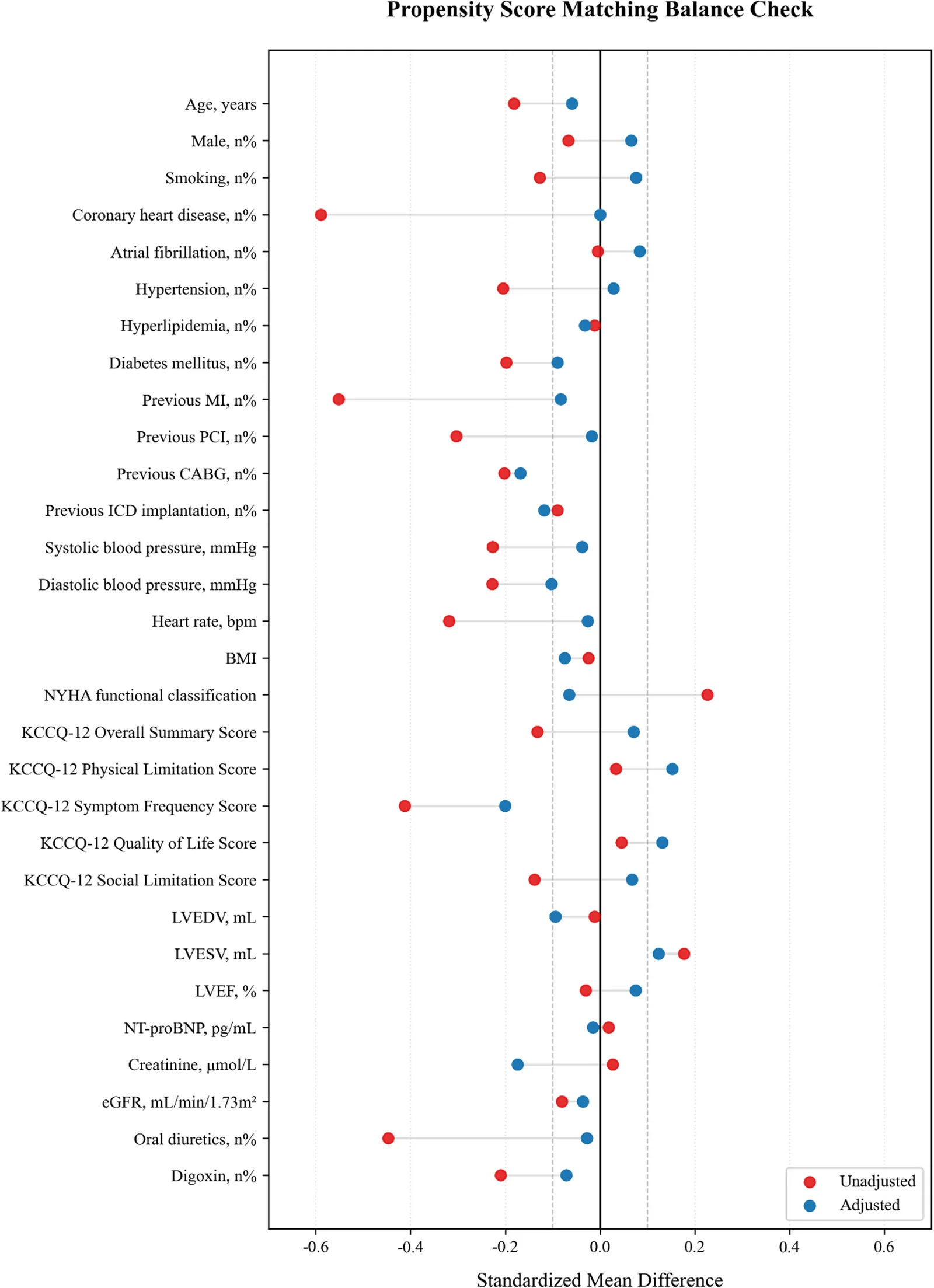
SMD for propensity score matching

After 1:1 PSM, 288 patients (144 per group) were balanced. Matching resulted in a balanced cohort of 288 patients (144 in each group). All demographic, clinical, laboratory, echocardiographic, and treatment-related variables were well balanced between the two groups (all *P* > 0.05) (Table 2; Fig. 3).

**Table 2:** Baseline clinical characteristics in the PSM cohort

	vericiguat	control	*P*
*n*	144	144	
Age, years	58.33 ± 11.47	59.01 ± 11.38	0.614
Male, *n*%	113 (78.47)	109 (75.69)	0.674
Smoking, *n*%	46 (31.94)	41 (28.47)	0.608
Coronary heart disease, *n*%	88 (61.11)	88 (61.11)	1.000
Atrial fibrillation, *n*%	11 (7.64)	8 (5.56)	0.636
Hypertension, *n*%	63 (43.75)	61 (42.36)	0.905
Hyperlipidemia, *n*%	34 (23.61)	36 (25.00)	0.891
Diabetes mellitus, *n*%	43 (29.86)	49 (34.03)	0.528
Previous MI, *n*%	30 (20.83)	35 (24.31)	0.573
Previous PCI, *n*%	27 (18.75)	28 (19.44)	1.000
Previous CABG, *n*%	0 (0.00)	2 (1.39)	0.498
Previous ICD implantation, *n*%	0 (0.00)	1 (0.69)	1.000
Systolic blood pressure, mmHg	120.68 ± 17.68	121.39 ± 19.96	0.750
Diastolic blood pressure, mmHg	74.66 ± 11.93	75.96 ± 13.33	0.385
Heart rate, bpm	75.33 ± 10.63	75.63 ± 11.88	0.826
BMI	24.24 ± 3.37	24.50 ± 3.59	0.530
NYHA functional classification			0.622
Ⅱ, *n*%	68 (47.22)	65 (45.14)	
Ⅲ, *n*%	65 (45.14)	65 (45.14)	
Ⅳ, *n*%	11 (7.64)	14 (9.72)	
KCCQ-12 Overall Summary Score	67.86 ± 12.35	66.81 ± 17.04	0.548
KCCQ-12 Physical Limitation Score	70.50 ± 14.73	67.92 ± 18.81	0.196
KCCQ-12 Symptom Frequency Score	79.21 ± 15.07	82.08 ± 13.44	0.089
KCCQ-12 Quality of Life Score	56.84 ± 19.06	54.05 ± 23.16	0.267
KCCQ-12 Social Limitation Score	64.89 ± 23.64	63.17 ± 27.16	0.567
LVEDV, mL	182.69 ± 60.09	199.80 ± 250.95	0.427
LVESV, mL	123.82 ± 98.63	114.51 ± 39.12	0.294
LVEF, %	35.77 ± 6.46	35.36 ± 6.13	0.581
LVEF ≤ 40%, %	112 (77.78%)	106 (73.61%)	0.492
NT-proBNP, pg/mL	1409.52 (425.83, 3724.86)	1631.58 (654.58, 3693.28)	0.236
Creatinine, µmol/L	82.56 ± 14.20	84.98 ± 13.50	0.140
eGFR, mL/min/1.73 m²	101.75 ± 14.43	102.24 ± 12.45	0.759
Oral diuretics, *n*%	69 (47.92)	71 (49.31)	0.906
Digoxin, n%	25 (17.36)	29 (20.14)	0.651

### Primary clinical outcomes in the PSM cohort

**Fig. 4:**
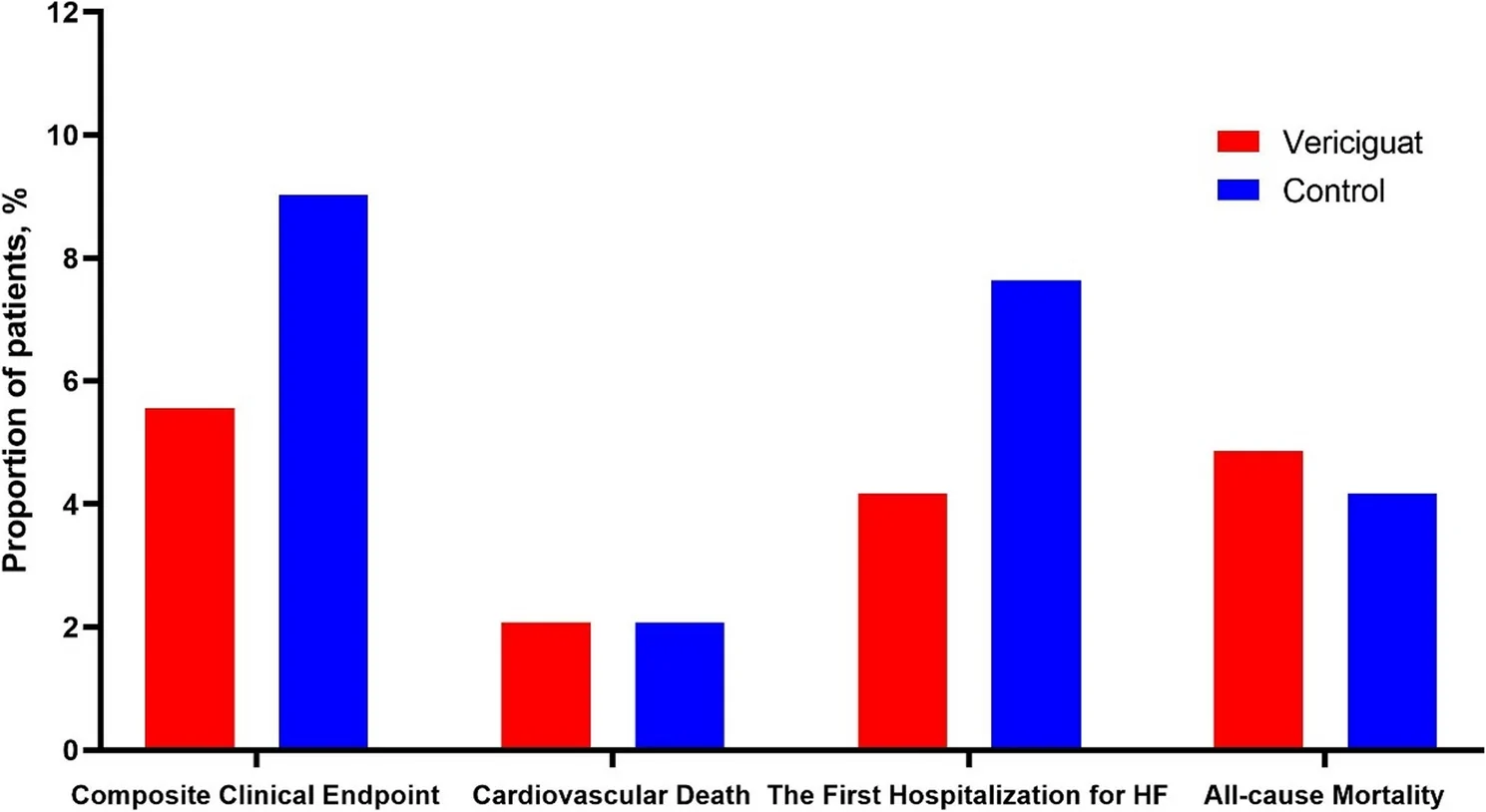
Clinical endpoints at 12-month in the PSM cohort. (Using the McNemar test.)

After 1:1 PSM, the primary composite endpoint of cardiovascular death or first HF hospitalization occurred in 8 vericiguat patients (5.56%) versus 13 control patients (9.03%), with no statistically significant difference (*P* = 0.365). First HF hospitalization rates were 4.17% vs. 7.64% (*P* = 0.317), cardiovascular death 2.08% vs. 2.08% (*P* = 1.000), and all‑cause mortality 4.86% vs. 4.17% (*P* = 1.000) (Fig. 4, Table S1).

**Fig. 5:**
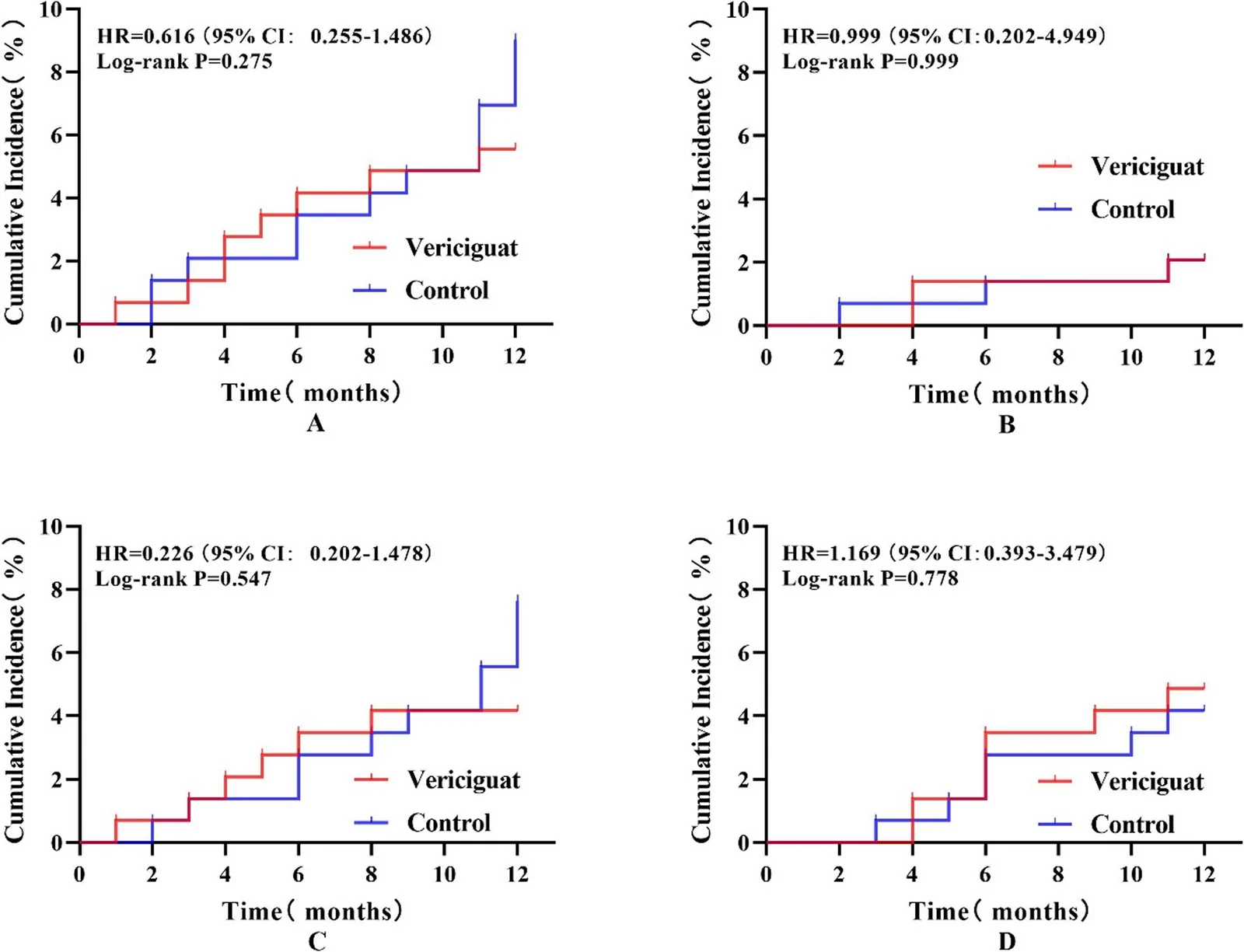
Kaplan-Meier curves in the PSM cohort. **A**: Kaplan-Meier curve for the composite clinical endpoint; **B**: Kaplan-Meier curve for the cardiovascular death; **C**: Kaplan-Meier curve for first HF hospitalization; **D**: Kaplan-Meier curve for all-cause mortality. Using the Kaplan-Meier method, and the stratified log-rank test

The corresponding hazard ratio for the composite endpoint was 0.616 (95% CI: 0.255–1.486, *P* = 0.275). For the individual components: cardiovascular death HR = 0.999 (95% CI: 0.202–4.949, *P* = 0.999), all-cause mortality HR = 1.169 (95% CI: 0.393–3.479, *P* = 0.778), first HF hospitalization HR = 0.226 (95% CI: 0.202–1.478, *P* = 0.547) (Fig. 5).

### NYHA functional class and quality of life in the PSM cohort

**Fig. 6:**
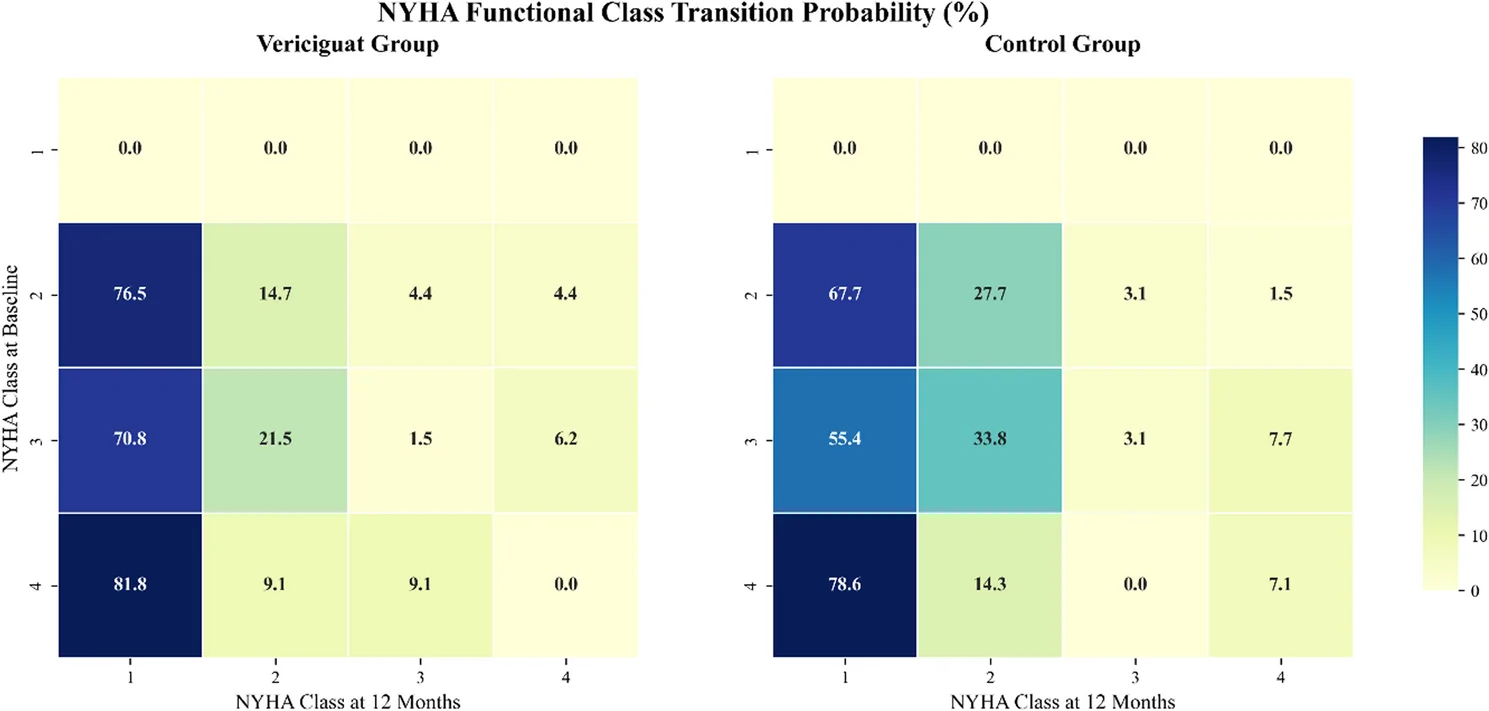
Cardiac function transition over 12 months in the PSM cohort

At 12 months, NYHA functional class distribution differed significantly between groups (*P* = 0.029). The vericiguat group had more class Ⅰ (76.39% vs. 63.19%) and fewer class Ⅱ (15.28% vs. 29.17%) patients (Table 3). Among baseline NYHA class Ⅱ patients, 76.5% in the vericiguat group improved to class Ⅰ versus 67.7% in controls. Among baseline NYHA class Ⅲ patients, 70.8% in the vericiguat group improved to class Ⅰ versus 55.4% in controls. Among baseline class Ⅳ patients, 81.8% improved to class Ⅰ versus 78.6% in controls (Fig. 6, Figure S1).

**Table 3:** NYHA functional classification at 12 months

	vericiguat	control	*P*
NYHA functional class, *n*%			0.029
Ⅰ	110 (76.39%)	91 (63.19%)	
Ⅱ	22 (15.28%)	42 (29.17%)	
Ⅲ	5 (3.47%)	4 (2.78%)	
Ⅳ	7 (4.86%)	7 (4.86%)	

**Fig. 7:**
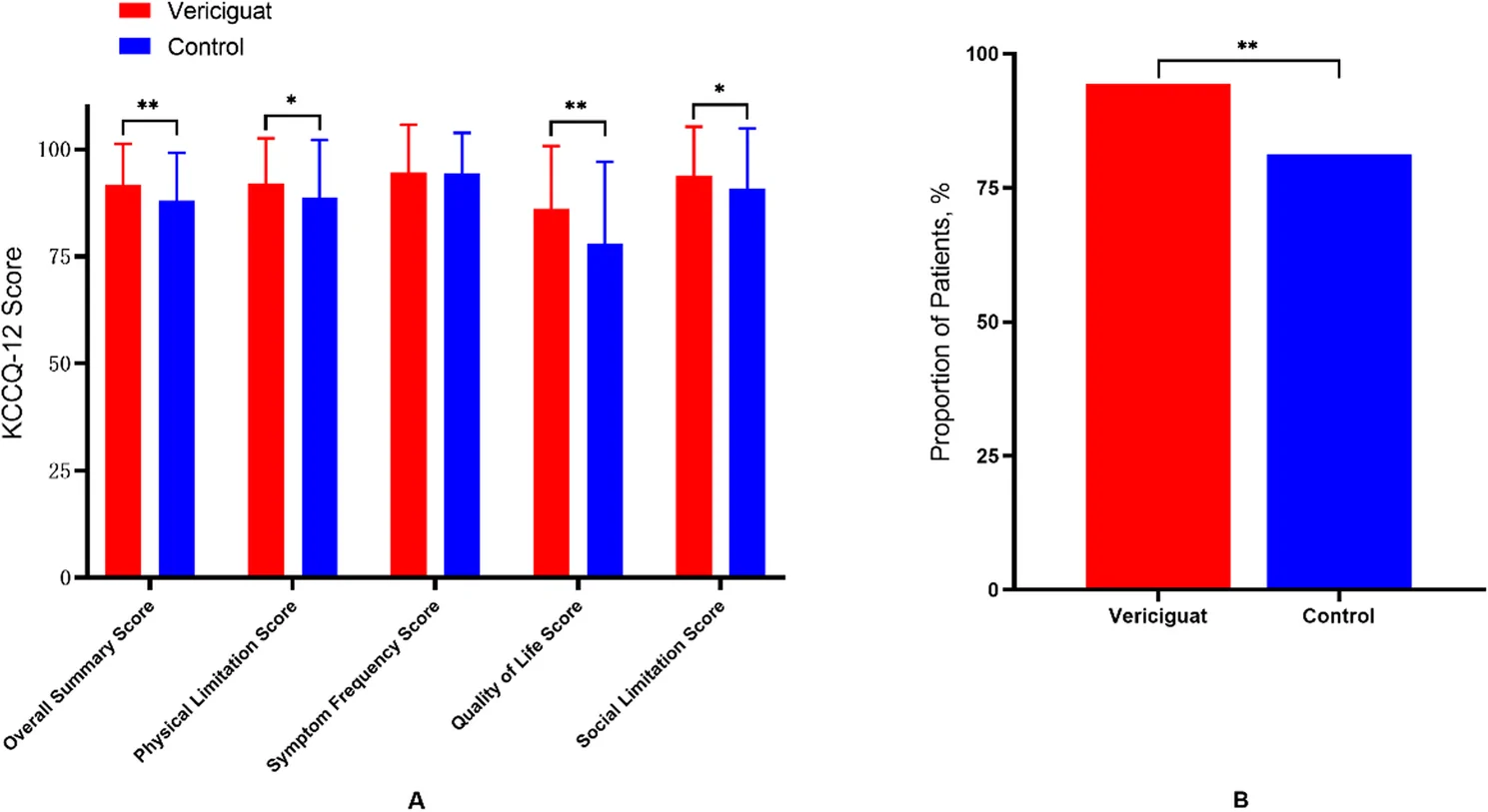
KCCQ-12 scores at 12-month in the PSM cohort. (A: KCCQ-12 scores of each dimension for both groups. Using paired t-tests; B: Proportion of patients with an improvement of more than 5 points in KCCQ-12 Overall Summary Score. Using the McNemar test. *P < 0.05, ‌**P < 0.01)

The vericiguat group showed significantly higher KCCQ-12 scores: Overall Summary Scores (91.61 ± 9.56 vs. 87.93 ± 11.25, *P* = 0.003), Physical Limitation Scores (91.90 ± 10.64 vs. 88.77 ± 13.38, *P* = 0.029), Quality of Life Scores (86.11 ± 14.61 vs. 77.94 ± 19.13, *P* < 0.001), and Social Limitation Scores (93.89 ± 11.39 vs. 90.74 ± 14.11, *P* = 0.038). No significant between-group difference was observed in Symptom Frequency Score (94.55 ± 11.17 vs. 94.29 ± 9.51, *P* > 0.05) (Fig. 7A, Table S3).

A higher proportion of vericiguat group patients achieved a ≥ 5-point improvement in KCCQ-12 Overall Summary Score (94.44% vs. 81.25%, *P* = 0.001), indicating that a treatment regimen initiated with standard quadruple GDMT combined with vericiguat leads to significantly greater improvement in health-related quality of life (Fig. 7B).

**Fig. 8:**
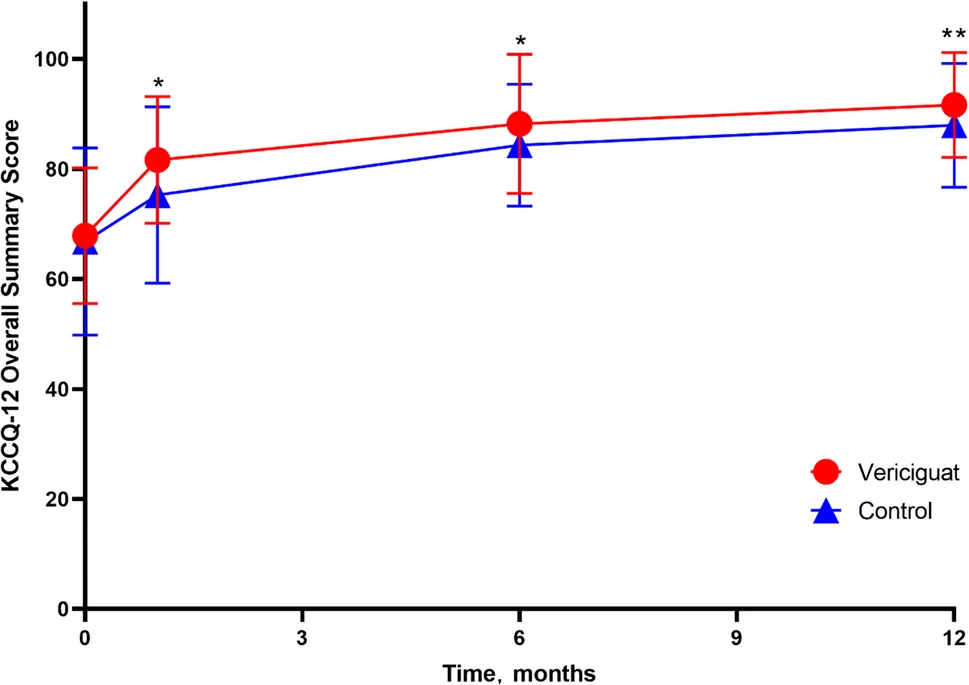
KCCQ-12 Overall Summary Score over 12 months in the PSM cohort. (*P < 0.05, ‌**P < 0.01. Using paired t-tests)

From month 1 onward, KCCQ-12 Overall Summary Scores were consistently higher in the vericiguat group than in the control group at 1 month (81.63 ± 11.52 vs. 75.24 ± 16.03, *P* = 0.045), 6 months (86.38 ± 9.54 vs. 81.89 ± 11.60, *P* = 0.034), and 12 months (91.61 ± 9.56 vs. 87.93 ± 11.25, *P* = 0.003). The between-group difference was most pronounced at 1 month, suggesting a potential early effect on quality-of-life measures; however, this observation is exploratory and requires confirmation in blinded randomized trials (Fig. 8).

### Subgroup analysis in the PSM Cohort

**Fig. 9:**
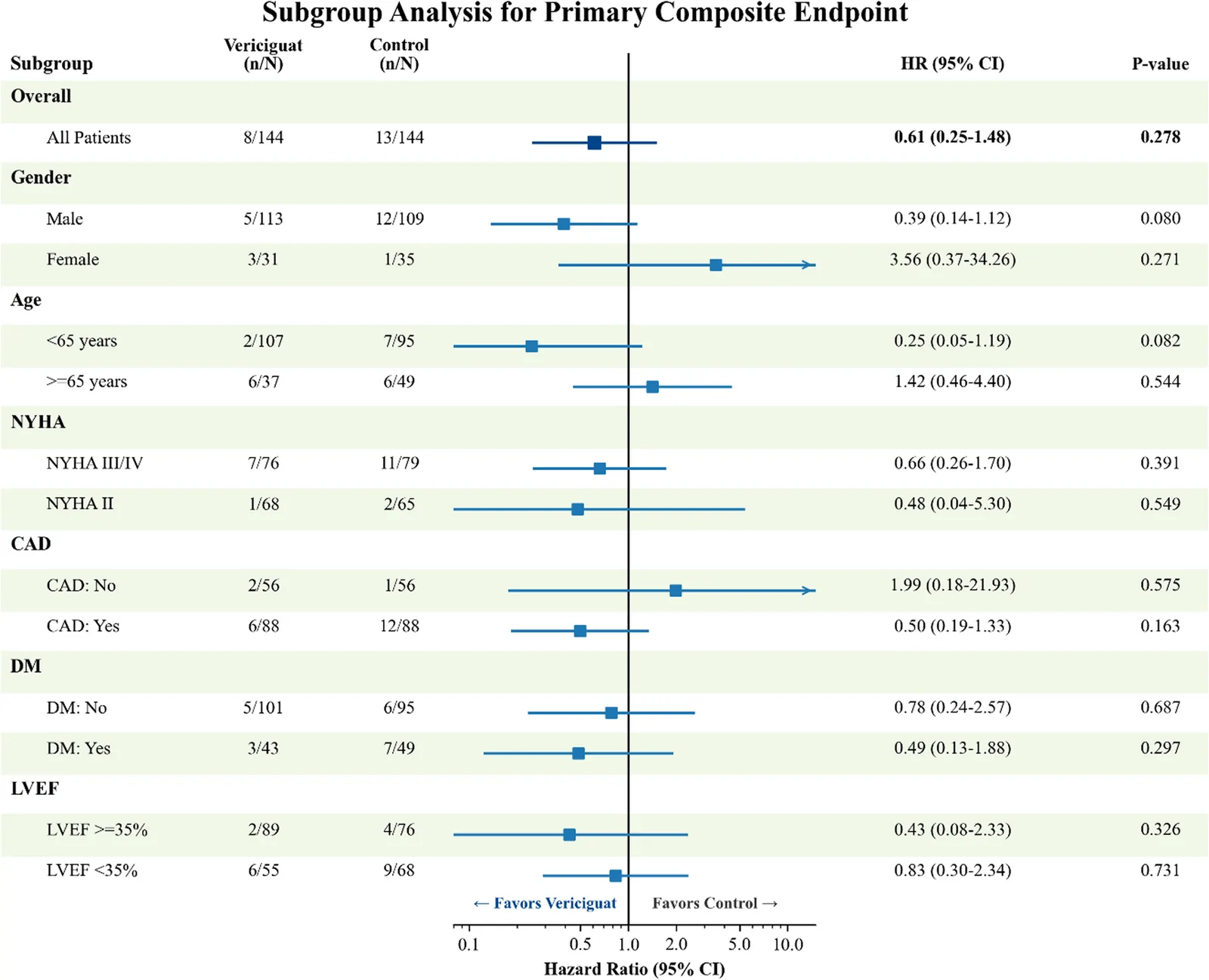
Forest plot of subgroup analysis in the PSM cohort. (CAD=coronary artery disease; DM=diabetes mellitus)

In the exploratory subgroup analysis of the primary composite endpoint, vericiguat showed a suggestive trend of risk reduction across the majority of subgroups, with HRs consistently below 1. The most favorable trends were observed in male patients (HR = 0.39, *P* = 0.080) and patients aged < 65 years (HR = 0.25, *P* = 0.082). Consistent with the overall population, no significant heterogeneity was detected, and there was no evidence of harm in any subgroup. However, due to the low number of events and wide confidence intervals in these subgroups, these findings should be considered exploratory and hypothesis-generating (Fig. 9).

### Safety profile in the PSM Cohort

**Fig. 10:**
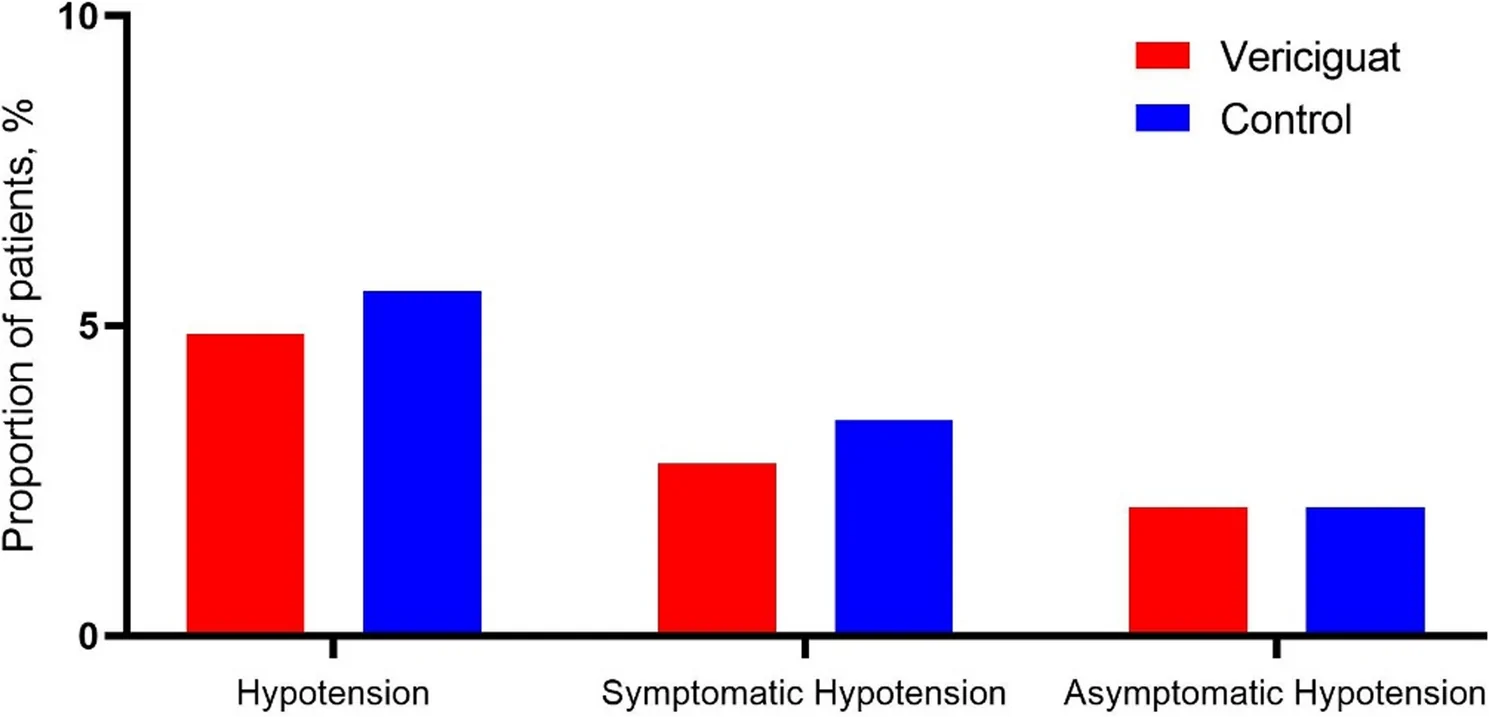
Incidence rates of hypotension over 12 months in the PSM cohort. (Using the McNemar test)

The incidence of hypotension, encompassing both symptomatic and asymptomatic events, was comparable between the two groups. Notably, vericiguat did not increase the risk of symptomatic hypotension (2.78% vs. 3.47%), demonstrating a favorable safety profile when administered alongside quadruple GDMT (Fig. 10). The incidence of dizziness and nausea did not differ significantly between the two groups. Syncope, renal events, and discontinuation due to adverse reactions were not reported in either group (Figure S2).

## Discussion

Vericiguat reactivates the compromised NO-sGC-cGMP signaling pathway—a key pathophysiological target not fully addressed by quadruple GDMT [15, 16]. Preclinical evidence has shown that vericiguat can repair cardiac microvascular endothelial damage, improve myocardial microcirculation, and reverse adverse ventricular remodeling [17]. Current international guidelines recommend vericiguat for high-risk patients with recent decompensation requiring hospitalization or intravenous diuretics [8, 10]. The therapeutic value of vericiguat in stable HFrEF patients without recent worsening HF remains underexplored.

In this propensity‑matched real‑world cohort of stable HFrEF patients (LVEF < 45%) receiving optimized quadruple GDMT, the addition of vericiguat did not significantly reduce the composite risk of cardiovascular death or first HF hospitalization at 12 months (HR 0.616, 95% CI 0.255–1.486; *P* = 0.275). The wide confidence interval crossing the null, combined with the low number of events (*n* = 21) and limited sample size after matching (*n* = 288), suggests that this nonsignificant result may reflect insufficient statistical power rather than an absence of treatment effect. A post-hoc power analysis indicated approximately 62% power to detect the observed hazard ratio. Therefore, no definitive conclusion regarding the effect of vericiguat on cardiovascular death or first HF hospitalization can be drawn from this study.

Exploratory analyses showed that vericiguat was associated with greater improvements in NYHA functional class and selected domains of the KCCQ-12 questionnaire, without increasing the risk of hypotension. These findings are hypothesis-generating and require confirmation in adequately powered randomized controlled trials before any inference regarding clinical benefit in stable HFrEF can be made.

Contemporary HFrEF management emphasizes early initiation of multi-pathway pharmacotherapy, with prompt GDMT uptitration being critical for improving long-term prognosis [18, 19]. Despite receiving standard quadruple GDMT, many patients remain at substantial risk [4]. Whether vericiguat can mitigate this residual risk in stable HFrEF populations remains to be established.

The VICTOR trial enrolled chronic HFrEF patients without recent worsening HF but had a key limitation: low proportion receiving standard quadruple GDMT (MRA < 80%, ARNI and SGLT2i < 60%) [11, 12]. Our study provides real-world data from a Chinese population on full quadruple GDMT, addressing an evidence gap regarding vericiguat in the context of contemporary optimized therapy. However, unlike VICTOR trial, our study was observational and underpowered for hard endpoints, precluding any causal inference.

Baseline data before PSM revealed that the vericiguat group had lower NYHA class Ⅱ proportion, higher NYHA class Ⅲ proportion, and lower KCCQ-12 Symptom Frequency Scores, indicating greater baseline symptom burden. After 1:1 PSM, the composite clinical endpoint did not differ significantly between groups, though the control group showed a numerical trend toward higher first HF hospitalization rates. At 12 months, NYHA class distribution differed significantly (*P* = 0.029): the vericiguat group had more NYHA class Ⅰ (76.39% vs. 63.19%) and fewer NYHA class Ⅱ (15.28% vs. 29.17%). Transitions analysis showed that among baseline NYHA class Ⅲ patients, 92.3% in the vericiguat group versus 89.2% in the control group improved to class Ⅰ/Ⅱ; among baseline NYHA class Ⅱ patients, improvement to class Ⅰ was 76.5% versus 67.7%. These NYHA improvements are exploratory observations that may reflect patient-reported functional status rather than objective clinical benefit, and their clinical significance requires prospective validation.

At 12 months, the vericiguat group exhibited significantly higher KCCQ-12 scores in Overall Summary Scores (91.61 ± 9.56 vs. 87.93 ± 11.25, *P* = 0.003), Physical Limitation Scores (91.90 ± 10.64 vs. 88.77 ± 13.38, *P* = 0.029), Quality of Life Scores (86.11 ± 14.61 vs. 77.94 ± 19.13, *P* < 0.001) and Social Limitation Scores (93.89 ± 11.39 vs. 90.74 ± 14.11, *P* = 0.038). No significant difference was observed in the Symptom Frequency domain, likely because all enrolled patients were in a clinically stable state with no recent decompensation, and optimal quadruple GDMT already controlled acute symptoms. While these KCCQ-12 differences reached statistical significance, they were not prespecified endpoints and should be interpreted as exploratory findings. The minimal clinically important difference (MCID) for the KCCQ may be less than the conventional 5-point threshold, a ≥ 5-point increase represents clinically meaningful improvement [20]. In this study, a ≥ 5-point improvement was observed in 94.44% of the vericiguat group versus 81.25% of the control group (*P* = 0.001). Dynamic analysis showed consistently higher scores in the vericiguat group from 1 month onward, with the most pronounced improvement in the first month. The observed early improvement in KCCQ-12 scores from month 1 onwards suggests a potential rapid effect on quality of life, but these patient-reported outcomes require confirmation in blinded randomized trials.

In the PSM cohort, cardiovascular death rate was identical between groups (2.08% vs. 2.08%) and first HF hospitalization rate was numerically lower in the vericiguat group (4.17% vs. 7.64%) though not statistically significant. The HR for the composite endpoint was consistent with the VICTOR trial’s primary result (HR = 0.93, 95% CI: 0.83–1.04; *P* = 0.22) [21–23]. Notably, VICTOR trial’s prespecified secondary analyses revealed significant benefits on cardiovascular death (HR 0.83, 95% CI 0.71–0.97; *P* = 0.020) and all-cause mortality (HR 0.84, 95% CI 0.74–0.97; *P* = 0.015) [21–23]. It remains to be determined whether similar benefits extend to stable HFrEF patients receiving optimized quadruple GDMT, highlighting the need for further investigation.

In exploratory subgroup analyses, HRs were generally below 1 across most subgroups, with numerically favorable trends in male patients (HR 0.39, *P* = 0.080) and patients aged < 65 years (HR = 0.25, *P* = 0.082). Consistent directional trends were observed in high-risk subgroups including those with coronary artery disease, diabetes mellitus, and NYHA functional class Ⅲ/Ⅳ, though none reached statistical significance due to limited sample sizes and low event rates. No subgroup exhibited an HR significantly > 1, there was no evidence of harm in any subgroup. However, due to the low number of events and wide confidence intervals in these subgroups. These subgroup findings are exploratory and underpowered to detect treatment-effect heterogeneity or definitively confirm safety. Therefore, they represent speculative hypotheses that require validation in larger, adequately powered trials.

A key clinical concern regarding vericiguat combined with quadruple GDMT is hypotension risk. Overall and symptomatic hypotension were comparable between groups, consistent with vericiguat’s established safety profile. Delayed GDMT initiation increases death risk even in low-risk HFrEF patients [24, 25]. And low systolic blood pressure is both a major barrier to GDMT dose uptitration and a poor prognostic predictor [26]. The VICTORIA trial found that systolic blood pressure nadirs within the first 8 weeks of vericiguat treatment and then returns to baseline levels, a pattern possibly due to progressive improvement in cardiac function and enhanced drug tolerance [27]. Our finding of blood pressure reduction did not differ significantly, supporting that vericiguat does not hinder GDMT uptitration. Moreover, baseline systolic blood pressure < 110 mmHg does not affect vericiguat’s efficacy or safety, with dose limitations primarily dictated by predefined titration protocols rather than drug intolerance [6, 27, 28]. Notably, vericiguat confers greater benefits in patients with lower baseline NT-proBNP (indicating early-stage HF) [29], which strongly supporting early combination with quadruple GDMT in HFrEF patients without recent worsening HF.

This study has several limitations. First, as an observational study without randomization or blinding, unmeasured confounders may remain, residual confounding bias cannot be completely eliminated despite 1:1 PSM. Second, statistical power was limited (288 patients, 21 composite endpoint events; HR 0.616, 95% CI: 0.255–1.486). The wide 95% CI which crosses the null suggests insufficient sample size, limiting precision and power. Thus, the non-significant composite clinical endpoint events may reflect insufficient statistical power rather than absence of treatment effect of vericiguat. Third, follow-up was limited to 12 months in stable chronic HF patients, precluding assessment of vericiguat’s long-term outcomes. Fourth, secondary outcomes are subjective and may be influenced by open-label, non-randomized design, differences in follow-up, and baseline symptom variations. Fifth, detailed dose titration and plasma concentration data were not collected, preventing dose-efficacy analysis. Sixth, serial echocardiographic parameters and NT-proBNP trajectories were not collected. Seventh, this single-center study in Chinese patients may limit generalizability.

Future multicenter randomized controlled trials with larger, more diverse populations, longer follow-up, and dose-response evaluation are needed to definitively evaluate vericiguat’s effects on composite clinical endpoint events and long-term prognosis in HFrEF patients without recent worsening HF.

## Conclusion

This propensity-matched observational study of stable HFrEF patients receiving optimized quadruple GDMT did not demonstrate a statistically significant reduction in cardiovascular death or first HF hospitalization with the addition of vericiguat. The study was underpowered for hard clinical endpoints, and the nonsignificant result does not exclude a potential treatment effect. Exploratory observations suggested potential associations with faster and greater improvements in health‑related quality of life and NYHA functional class, without increasing symptomatic hypotension. These hypothesis‑generating findings warrant confirmation in adequately powered, multicenter, randomized controlled trials with longer follow‑up.

## Supplementary Information

Supplementary Material 1.

## Data Availability

The datasets used and analyzed during the current study are available from the corresponding author upon reasonable request.

## Abbreviations

ACEI: Angiotensin-converting enzyme inhibitor
ARB: Angiotensin Ⅱ receptor blocker
ARNI: Angiotensin receptor–neprilysin inhibitor
BB: Beta-blocker
BMI: Body mass index
CABG: Coronary artery bypass grafting
ChiCTR: Chinese Clinical Trial Registry
CI: Confidence interval
cGMP: Cyclic guanosine monophosphate
DBP: Diastolic blood pressure
eGFR: Estimated glomerular filtration rate
GDMT: Guideline-directed medical therapy
HF: Heart failure
HFrEF: Heart failure with reduced ejection fraction
HR: Hazard ratio
ICD: Implantable cardiovertor-defibrillator
KCCQ-12: Kansas City Cardiomyopathy Questionnaire-12
LVAD: Left ventricular assist device
LVEDV: Left ventricular end-diastolic volume
LVEF: Left ventricular ejection fraction
LVESV: Left ventricular end-systolic volume
MI: Myocardial infarction
MRA: Mineralocorticoid receptor antagonist
NO: Nitric oxide
NT-proBNP: N-terminal pro–B-type natriuretic peptide
NYHA: New York Heart Association
PCI: Percutaneous coronary intervention
PDE-5: Phosphodiesterase-5
PSM: Propensity score matching
SBP: Systolic blood pressure
Scr: Serum creatinine
SGLT2i: Sodium-glucose cotransporter-2 inhibitor
sGC: Soluble guanylate cyclase
SMD: Standardized mean difference
STROBE: STrengthening the Reporting of OBservational studies in Epidemiology
