# Supplementary material for: Vericiguat for heart failure with reduced ejection fraction on quadruple therapy without recent worsening heart failure: an exploratory real-world cohort study
Source: BMC Cardiovasc Disord. 2026 Jul 21;26:948. doi: 10.1186/s12872-026-06319-1 (PMC13685388; doi:10.1186/s12872-026-06319-1)
Supplement: Supplementary file 1 [file 12872_2026_6319_MOESM1_ESM.docx]

**Table S1.** Clinical endpoint at 12 months in the PSM cohort

|  | **Vericiguat** | **Control** | **P** |
| --- | --- | --- | --- |
| Composite clinical endpoint | 8 (5.56%) | 13 (9.03%) | 0.365 |
| Cardiovascular death | 3 (2.08%) | 3 (2.08%) | 1.000 |
| First hospitalization for HF | 6 (4.17%) | 11 (7.64%) | 0.317 |
| All-cause mortality | 7 (4.86%) | 6 (4.17%) | 1.000 |

**Table S2.** NYHA functional classification at 12 months before PSM

|  | **Vericiguat** | **Control** | **P** |
| --- | --- | --- | --- |
| NYHA functional class, n% |  |  | 0.036 |
| Ⅰ | 145 (75.13%) | 159 (65.70%) |  |
| Ⅱ | 43 (22.28%) | 76 (31.41%) |  |
| Ⅲ | 5 (2.59%) | 6 (2.48%) |  |
| Ⅳ | 0 | 1 (0.41%) |  |

 **Figure S1.** Evolution of NYHA functional classification at 12 months in the PSM cohort

**Table S3.** KCCQ-12 scores at 12 months in the PSM cohort

|  | **Vericiguat** | | **Control** | **P** |
| --- | --- | --- | --- | --- |
| KCCQ-12 Physical Limitation | | 91.97 ± 10.90 | 89.01 ± 13.62 | 0.048 |
| KCCQ-12 Symptom Frequency | | 94.48 ± 11.45 | 94.25 ± 9.71 | 0.857 |
| KCCQ-12 Quality of Life | | 86.25 ± 14.97 | 77.72 ± 19.52 | <0.001 |
| KCCQ-12 Social Limitation | | 93.92 ± 11.68 | 90.70 ± 14.42 | 0.043 |
| KCCQ-12 Total Score | | 91.66 ± 9.80 | 87.92 ± 11.49 | 0.004 |
| Change in KCCQ-12 Physical Limitation | | 21.28 ± 17.28 | 21.10 ± 21.40 | 0.938 |
| Change in KCCQ-12 Symptom Frequency | | 15.19 ± 18.65 | 12.09 ± 15.58 | 0.136 |
| Change in KCCQ-12 Quality of Life | | 29.68 ± 23.38 | 23.94 ± 29.45 | 0.075 |
| Change in KCCQ-12 Social Limitation | | 29.30 ± 25.47 | 27.38 ± 28.42 | 0.556 |
| Change in KCCQ-12 Total Score | | 23.86 ± 14.94 | 21.13 ± 18.80 | 0.183 |

**Figure S2.** Drug-related adverse events in the PSM cohort

(Using the McNemar test)
